# Rosmarinic acid potentiates carnosic acid induced apoptosis in lung fibroblasts

**DOI:** 10.1371/journal.pone.0184368

**Published:** 2017-09-06

**Authors:** Sana Bahri, Frédérique Mies, Ridha Ben Ali, Mona Mlika, Saloua Jameleddine, Kathleen Mc Entee, Vadim Shlyonsky

**Affiliations:** 1 Department of physiology, University of Tunis El Manar, La Rabta, Tunis, Tunisia; 2 Department of Physiology and Pharmacology, Université libre de Bruxelles, Brussels, Belgium; 3 Department of Physiopathology, Food and Biomolecules (LR-17-ES-03), Technology Center of Sidi Thabet, University of Manouba, Tunis, Tunisia; 4 Department of Experimental Medicine, University of Tunis El Manar, La Rabta, Tunis, Tunisia; 5 Department of Anatomy and Pathology, Abderhaman Mami Hospital, Ariana, Tunisia; University of South Alabama Mitchell Cancer Institute, UNITED STATES

## Abstract

Pulmonary fibrosis is characterized by over-population and excessive activation of fibroblasts and myofibroblasts disrupting normal lung structure and functioning. Rosemary extract rich in carnosic acid (CA) and rosmarinic acid (RA) was reported to cure bleomycin-(BLM)-induced pulmonary fibrosis. We demonstrate that CA decreased human lung fibroblast (HLF) viability with IC_50_ value of 17.13±1.06 μM, while RA had no cytotoxic effect. In the presence of 50 μM of RA, dose-response for CA shifted to IC_50_ value of 11.70±1.46 μM, indicating synergic action. TGFβ-transformed HLF, rat lung fibroblasts and L929 cells presented similar sensitivity to CA and CA+RA (20μM+100μM, respectively) treatment. Rat alveolar epithelial cells died only under CA+RA treatment, while A549 cells were not affected. Annexin V staining and DNA quantification suggested that HLF are arrested in G0/G1 cell cycle phase and undergo apoptosis. CA caused sustained activation of phospho-Akt and phospho-p38 expression and inhibition of p21 protein.Addition of RA potentiated these effects, while RA added alone had no action.Only triple combination of inhibitors (MAPK-p38, pan-caspase, PI3K/Akt/autophagy) partially attenuated apoptosis; this suggests that cytotoxicity of CA+RA treatment has a complex mechanism involving several parallel signaling pathways. The *in vivo* antifibrotic effect of CA and RA was compared with that of Vitamine-E in BLM-induced fibrosis model in rats. We found comparable reduction in fibrosis score by CA, RA and CA+RA, attenuation of collagen deposition and normalization of oxidative stress markers. In conclusion, antifibrotic effect of CA+RA is due to synergistic pro-apoptotic action on lung fibroblasts and myofibroblasts.

## Introduction

Idiopathic pulmonary fibrosis (IPF) is the most common and predominantly lethal form of the idiopathic interstitial pneumonias, with an associated median survival of only 2 to 3 years [1]. The pathobiological mechanisms underlying the development of IPF are highly complex. Recurring damage to the epithelium results in an abnormal wound healing response characterized by dysregulated epithelial–mesenchymal crosstalk and the accumulation of myofibroblasts [2]. These cells synthesize excessively all the components of the extracellular matrix and thus replace the normal structure of the lung leading to a functional impairment that facilitates the installation of fibrosis. Therefore, myofibroblasts and type II alveolar epithelial cells are considered as principal players in this disease [3].

Despite the progress that has been made to understand the pathophysiology of IPF, pirfenidone and nintedanib remain currently the only therapeutic agents approved worldwide. Hence, the development of new treatment modalities is critically important to target more than one of the profibrotic pathways associated with the complex pathogenesis of IPF. For a long time, the use of medicinal plants was the principal remedy of many diseases by our ancestors. Nowadays, the development of pharmaceutical industry allowed the direct use of natural bioactive substances extracted from plants with a high therapeutic power, which maintains the phytotherapy alive until today.

*Rosmarinus officinalis*is very reputed in traditional medicine. Its biological properties are due in large part to phenolic constituents, especially rosmarinic and carnosic acids. This latter is the main phenolic diterpene that was reported to have numerous biological properties, acting like an antioxidant [4], an antitumor [5] an antimicrobial [6], an antifungal [7], an antiangiogenic [8], an anti-inflammatory [9] and a chemoprotective [10] agent. Rosmarinic acid also possesses many biological activities, mainly anti-inflammatory [11], anti-oxidative [12, 13], anti-apoptotic [14], antifibrotic[15] and neuroprotective [16] activities. The capacity of these two molecules to modulate several processes has been suggested to be beneficial for IPF treatment and we have shown recently that rosemary leaves extract has prophylactic and curative effect in bleomycin-induced model of pulmonary fibrosis in rats [17]. Therefore, in the current study we investigated the cellular and molecular effects of these molecules *in vitro* on human and rat lung fibroblasts, on rat type II pneumocytes, on A549 cells and on L929 cells and *in vivo* in an experimental model of pulmonary fibrosis induced by bleomycin in rats.

## Materials and methods

### Ethics statement

For in vitro study, the experiments were performed in accordance with Animal care ethics committee approval (Comité d’Ethique ULB–reference 442N) in conformity with NIH guideline (National Research Council, 1985). Nembutal anesthesia followed by exsanguination.For in vivo study, all experiments were performed according to the recommendations of the ethic committee of Tunis University for care and use of animals in conformity with NIH guideline (National Research Council, 1985). Pentobarbital anesthesia.

### Reagents

Carnosic acid and rosmarinic acids used *in vitro* were obtained from Sigma-Aldrich. For the *in vivo* study, these molecules were purchased from Santa Cruz Biotechnology Inc. BIRB796 was purchased from Santa Cruz Biotechnology. JNK inhibitor II and PD98059 were from Merck-Millipore. All other reagents and inhibitors were obtained from Sigma-Aldrich (Leuven, Belgium).

### Cell cultures

#### Human lung fibroblasts

Primary human lung fibroblasts (HLF) were purchased from Lonza and cultured in FGM-2 culture medium (Lonza, Verviers, Belgium) supplemented with BulletKit (CC-3132; Lonza) and 2% fetal bovine serum (FBS) at 37°C in the presence of 5% CO_2_. The 70–80% confluent cell culture flasks were passaged at a 1:3 ratio and used for up to eight passages. Before each of the tests cited below, cells were washed, detached using trypsin-EDTA 0.05%, treated with trypsin inhibitor to stop the reaction, counted using Burker cell, and centrifuged 5min at 300*g*. Culture medium was added and cells were seeded at appropriate density on the plates for each experiment.

#### Rat lung fibroblasts

Primary rat lung fibroblasts (RLF) were purchased from Cell Applications (San Diego, CA, USA) and cultured in RFGM medium (Rat Fibroblast Growth Medium, R116-500, Cell Applications) in a humidified incubator at 37°C and 5% CO_2_. Cells were passaged using 0.25% trypsin-EDTA after reaching a maximal confluence of 80%. These primary rat lung fibroblasts were used up to passage 5.

#### L929 and A549 cells

L929 fibroblasts and A549 alveolar epithelial cells were purchased from the European Collection of Cell Cultures through Sigma-Aldrich and cultured in DMEM supplemented with 10% fetal calf serum, 1% penicillin-streptomycin in a humidified incubator at 37°C and 5% CO_2_. No additional cell line authentication has been carried out. L929 cells were passaged at 1:5 ratio using 0.25% trypsin-EDTA after reaching a maximal confluence of 80% and were used for up to six consecutive passages. A549 were passaged at 1:10 ratio using 0.25% trypsin-EDTA after reaching full confluence.

#### Isolation and culture of rat alveolar type II cells

The alveolar type II cell culture was performed as described previously [18]. Rats were maintained under conditions of unlimited access to food and water and according to the guidelines issued by the local animal care ethics committee (Comité d’Ethique–reference 442N) in conformity with NIH guideline (National Research Council, 1985). Briefly, male Wistar rats were anesthetized with Nembutal and killed by exsanguination. Lungs were excised, trachea was catheterized, and the pulmonary vasculature was perfused via the pulmonary artery with *solution II* (140 mM NaCl, 5 mM KCl, 2.5 mM Na_2_HPO_4_, 10 mM HEPES, 1.3 mM MgSO_4_, and 2.0 mM CaCl_2_; pH 7.4) to remove the blood. The air spaces were then washed with *solution I* (140 mM NaCl, 5 mM KCl, 2.5 mM Na_2_HPO_4_, 6 mM glucose, 0.2 mM EGTA, and 10 mM HEPES; pH 7.4) to remove free, nonepithelial cells. Elastase solution (1 mg/ml dissolved in *solution II*) was instilled in the air space and lungs were incubated at 37°C for 10 min. The elastase instillation was repeated for another 10 min, and the large airways and heart were removed. The remaining lung tissue was then minced in 5 ml FBS (to stop elastase reaction) and in DNase IV (1mg/ml, 4 ml in 0.9% NaCl).The minced lungs were filtered through gauze then through 100 μm and 40 μm cell strainers, and the cell suspension was collected. The suspension was centrifuged and resuspended in AT II culture medium (DMEM with 10% heat-inactivated FBS, 4 mM glutamine, 1% penicillin/streptomycin, and 2.5 μM amphotericin B) and plated on untreated Petri dishes coated with IgG. The plates were incubated for 1 hour to allow nonepithelial cells such as macrophages and fibroblasts to adhere to the IgG. Non-attached cells were collected, centrifuged and used immediately.

### Cell growth / cytotoxicity test

Fibroblast growth and viability was studied using the Cell Counting Kit-8 (CCK-8). It consists in the observation of the living cell for 48 hours (cell growth assay) and for 24 hours (cytotoxicity assay) in the presence or absence of tested molecules. This colorimetric assay is based on the use of the cellular dehydrogenase substrate, WST-8 (2-(2-methoxy-4-nitrophenyl)-3-(4-nitrophenyl)-5-(2,4-disulfophenyl)-2H tetrazolium monosodium salt), which is reduced following its addition to the cells to yield a yellow product, the formazan. The formazan is detected at 450 nm and the optical density is directly proportional to the number of living cells in the culture wells. A minimum of six wells were seeded for each experimental condition.

### Cell migration test

Cell migration was studied using Boyden chambers (Tebu-Bio, Boechout, Belgium). This system consists of a 96 well plate filter inserts. Cells (5.10 ^3^) labeled with calcein-AM were loaded into the upper compartment (0.082 cm^2^ growing area) in the presence of the testing molecules and other factors added to the upper and lower compartment. After 6h incubation at 37°C, 5% CO_2_, fluorescent cells that migrated to the lower surface of the insertion filter were examined under inverted microscope, while the filter blocked the transmission of light from the upper compartments. A low power field view of the filter representing 36% of the surface was taken using CCD camera and cells were counted by two operators. Cell migration is expressed as a percentage of seeded cells; 8 wells were seeded for each experimental condition.

### Apoptosis test

Dual staining with annexin V conjugated to fluorescein-isothiocyanate (FITC) and with 7-amino-actinomysin (7-AAD) was performed using the commercially available kit (Muse Annexin V and Dead Cell Kit; Merck KGaA, Darmstadt, Germany). Briefly, cells were incubated for 24h at 37°C, 5% CO2, in the presence of testing substances in 6-well plates, then harvested and centrifuged for 5min at 300g. Cell pellet was resuspended in PBS containing 1% FBS and 100 μl of the cell suspension was added to an equal volume of the fluorescent reagent. The percentage of live cells (annexin V negative and 7AAD negative), early apoptotic cells (annexin V positive and 7AAD negative), late apoptotic cells (annexin V positive and 7AAD positive) and necrotic cells (annexin V negative and 7AAD positive) was acquired and analyzed by Muse^TM^ cell analyzer after 20 min incubation at room temperature in accordance with the manufacturer’s guidelines.

### Cell cycle test

The study of the effect of rosmarinic acid and carnosic acid on the cell cycle progression was performed using a nuclear dye, propidium iodide, to distinguish cells in different cell cycle stages depending on the amount of cellular DNA. Briefly, cells were incubated with the molecules alone or in combination for 24 h. Both floating and adherent cells were collected and centrifuged at 300g for 5 minutes. The pellet was washed and suspended in 100 μl of PBS, added to 1 ml of ice cold 70% ethanol and stored at -20°C. After 3 hours, cells were centrifuged at 300 g for 5 minutes, washed once with PBS and incubated with the fluorescent reagent for 30 min. The percentage of the population in the G_0_/G_1_, S and G_2_/M phases was measured using the Muse^TM^ Cell Analyser.

### Western blot analysis

HLF cells were cultured to 75% confluence. CA and RA alone or in combination (RC) were added to the medium and cells were incubated with these molecules for 30 min, 2 hours and 24 hours. Cell extracts were prepared by washing cultures with PBS and suspending in lysis buffer [20 mM Tris-HCl (pH 7.4), 150 mM NaCl, 1% NP-40, 1 mM EGTA, 1 mM EDTA] containing protease inhibitors (Roche) on ice. Cell extracts were sonicated for 2x15 seconds and centrifuged at 3000g for 10 min and the supernatant was used for western blotting. Protein concentration was determined using Advanced Protein kit (Sigma-Aldrich). Sample buffer was added to the cell lysate, and the mixed samples were boiled for 1 min at 95°C. Proteins were separated in 4–12% SDS-PAGE and transferred onto nitrocellulose membranes. Membranes were blocked for 60 min at room temperature in corresponding blocking buffer in TBS-T, followed by incubation with primary antibodies overnight at 4°C. The membranes were then washed three times for 10 min in TBS-T and incubated with horseradish peroxidase-conjugated secondary antibodies. Immunoreactive bands were detected using an enhanced chemiluminescence western blotting kit (ThermoScientific 3747N. Meridian Rd Rockford, IL61101, USA). Antibodies used were: rabbit anti-p38 (#9212, Cell Signaling), rabbit anti-phospho-p38 (#4511, Cell Signaling), rabbit anti-panAkt (#4691, Cell Signaling), rabbit anti-phosphoAkt (#4060, Cell Signaling), mouse anti-p21WAF/Cip (#P1484, Sigma-Aldrich), mouse anti-γ-tubulin (#T5326, Sigma-Aldrich), ImmunoPure® donkey anti-rabbit HRP-conjugated (H+L, ThermoScientific) and Pierce® goat anti-mouse HRP-conjugated (H+L, ThermoScientific). Prior to immunoblotting, protein loading level was routinely verified using Pierce^TM^ reversible protein stain kit (#24580, ThermoScientific). Accordingly, whole lane densitometry was performed.

### Animal model of bleomycin-induced lung fibrosis

Eighty Wistar adult male rats (160–200 g) were procured form Pasteur Institute, Tunis, Tunisia and fed with a standard laboratory food and water *ad libitum*. Rats were maintained in animal housing at a controlled temperature (22±2°C) with a 12 h light-dark cycle. All experiments were performed according to the recommendations of the ethic committee of Tunis University for care and use of animals in conformity with NIH guideline (National Research Council, 1985). Bleomycin (BLM) rat model of lung fibrosis as reported earlier [19] was performed. All rats underwent anesthesia by intraperitoneal injection of 100 μg/g of pentobarbital sodium solution (Sandoz Laboratory, France). Each anesthetized rat was immediately fixed on a gallows. Induction of fibrosis was done by an intra-tracheal injection of 4 mg/kg body weight (bw) of bleomycin sulfate solution (Bleomycin®, Laboratories Aventis, France).

#### Experimental design

Rats were divided into 10 groups of 8 animals each. Group I received a single intra-tracheal injection of normal saline (control group). Group II received a single intratracheal instillation of bleomycin (4 mg/kg bw*)* (rat model of lung fibrosis: BLM group). Group III received a daily intraperitoneal injection of RA (5 mg/kg bw) for 2 weeks (RA group). Group IV received a single intra-tracheal instillation of bleomycin (4 mg/kg bw) and a daily intraperitoneal injection of RA (5 mg/kg bw) that started on the third day after fibrosis induction and lasted for 2 weeks (BLM/RA group). Group V received a daily intraperitoneal injection of CA (3 mg/kg bw) for 2 weeks (CA group). Group VI received a single intra-tracheal instillation of bleomycin (4 mg/kg bw) and a daily intraperitoneal injection of RA (5 mg/kg bw) that started on the third day after fibrosis induction and lasted for 2 weeks (BLM/RA group). Group VII received a daily intraperitoneal injections of CA (3 mg/kg bw) and RA (5mg/kgbw) for 2 weeks (RA/CA group). Group VIII received a single intra-tracheal instillation of bleomycin (4 mg/kg bw) and a daily intraperitoneal injections of CA (3 mg/kg bw) and RA (5mg/kgbw) that started from the third day after fibrosis induction and lasted for 2 weeks (BLM/RA/CA group). Group IX received a daily intraperitoneal injections of vitamin E (300 mg/kg bw) for 2 weeks (Vit E group).Group X received a single intra-tracheal instillation of bleomycin (4 mg/kg bw) and a daily intraperitoneal injection of vitamin E (300 mg/kg bw) that started from the third day after fibrosis induction and lasted for 2 weeks (BLM/Vit E group). The intratracheal instillation of BLM causes a direct initial damage to alveolar epithelial cells, followed by the development of neutrophilic and lymphocytic pan-alveolitis within the first week [20]. The inflammatory reaction is triggered within the first days of this week, which is detected by the increased recruitment of perivascular and peribronchial lymphocytes in the lung [21]. Therefore, we chose the 3rd day after fibrosis induction for the start of the curative treatment.

#### Sample collection

At the end of the experiment, rats were anesthetized with urethane (40 mg/kg) and then exsanguinated by incision of abdominal aorta. The left lung was fixed for histological study and the right lung was weighted, homogenized in phosphate buffer saline and centrifuged for the measurement of oxidative stress indicators. The supernatant was stored at -80°C for eventual assays. Total soluble proteins level was evaluated according to the Biuret method [22].

#### Body weight determination

The body weight of rats was measured at the beginning and at the end of the experiment. The percentage of body weight gain in each group was compared and calculated by the following manner: [(final weight—initial weight) / initial weight] × 100.

### Hydroxyproline quantification

Hydroxyproline was measured as described by Reddy and Enwemeka [23]. Briefly, 1 g of lung tissue was homogenized in 1 ml of distilled water followed by hydrolysis in 2N NaOH in autoclave at 120°C for 20 min. Next, 50 μl of hydrolysate was oxidized at room temperature for 25 min by the addition of 450 μl of 56 mM Chloramine T. Then 500 μl of Ehrlish‘s aldehyde reagent (15g of dimethylaminobenzaldehyde dissolved in n-propanol/perchloric acid, 2:1 v/v) was added and the chromophore was developed by incubating the samples at 65°C for 20 min. The absorbance was measured at 550 nm and hydroxyproline content was expressed as mg/g of lung tissue.

### Histological study

For histological investigation, rat lungs were perfused and immersed in the fixative solution (10% neutral-buffered formalin) for 3 days, dehydrated through graded series of ethanol bath, embedded in paraffin, cut into 4 μm thick sections and finally stained with hematoxylin-eosin (H&E) or with Masson’s trichrome. In order to assess the severity and the extension of pulmonary lesions, we used a blinded semi quantitative scoring system to evaluate the level of fibrosis in lung parenchyma as described previously [24].This considers the following grades: Grade 0 = “normal lung”; Grade 1 = “minimal fibrous thickening of alveolar or bronchial walls”; Grades 2 to 3 = “moderate thickening of walls without obvious damage to lung architecture”; Grades 4 to 5 = “increased fibrosis with definite damage to lung architecture and formation of fibrous bands or small fibrous mass”;Grades 6 to 7 = “severe distortion of structure and large fibrous areas”, “honeycomb lung” was placed in this category; Grade 8 = “total fibrotic obliteration of the field”.

### Lipid peroxidation

Lipid peroxidation was assayed by the detection of thiobarbituric acid (TBA) reactive products as described previously [25]. Reaction with TBA can indicates the presence of small amounts of lipid peroxides, and more particularly the free malondialdehyde (MDA) produced during the oxidative breakdown of lipids and polyunsaturated fatty acids. Briefly, we incubated both lung supernatant and sodium phosphate buffer at 37°C for 1 h and the mixture was centrifuged after being precipitated with 10% TCA (trichloroacetic acid). Then we added 1% TBA to the supernatant and placed the mixture in the boiling water for 15 min. The absorbance was read at 532 nm and MDA level was expressed in nmol/mg protein using molar extinction coefficient of 156000 M^−1^ cm^−1^.

### Catalase and superoxide dismutase activity

Catalase activity (CAT) was determined as described previously [26]. Briefly, the assay mixture included 0.019 M H_2_O_2_, 0.05 M phosphate buffer (pH = 7) and 0.03 mL lung extract. CAT activity was calculated in terms of μmole of H_2_O_2_ consumed/minute/mg of protein. Superoxide dismutase (SOD) activity was evaluated as described previously [27], based on the inhibition of the auto-oxidation of epinephrine to adenochrome in the presence of SOD at pH = 10,2. Briefly, 20 μl epinephrine (5mg/ml) was added to the assay mixture containing 10μl bovine catalase (0.4 U/μl), and 62.5 mM sodium carbonate-sodium bicarbonate buffer. One unit of SOD is defined as the enzyme required to inhibit 50% of adenochrome generation. The absorbance was recorded at 480 nm.

### Statistical analysis

Data are presented as Mean±SD. For all *post-hoc* multiple comparisons, statistical significance was determined by ANOVA followed by Sidak's test. A *p*-value of 0.05 or less was considered as statistically significant.

## Results

### Effects of CA, RA, and CA + RA on HLF cells viability

**Fig 1:**
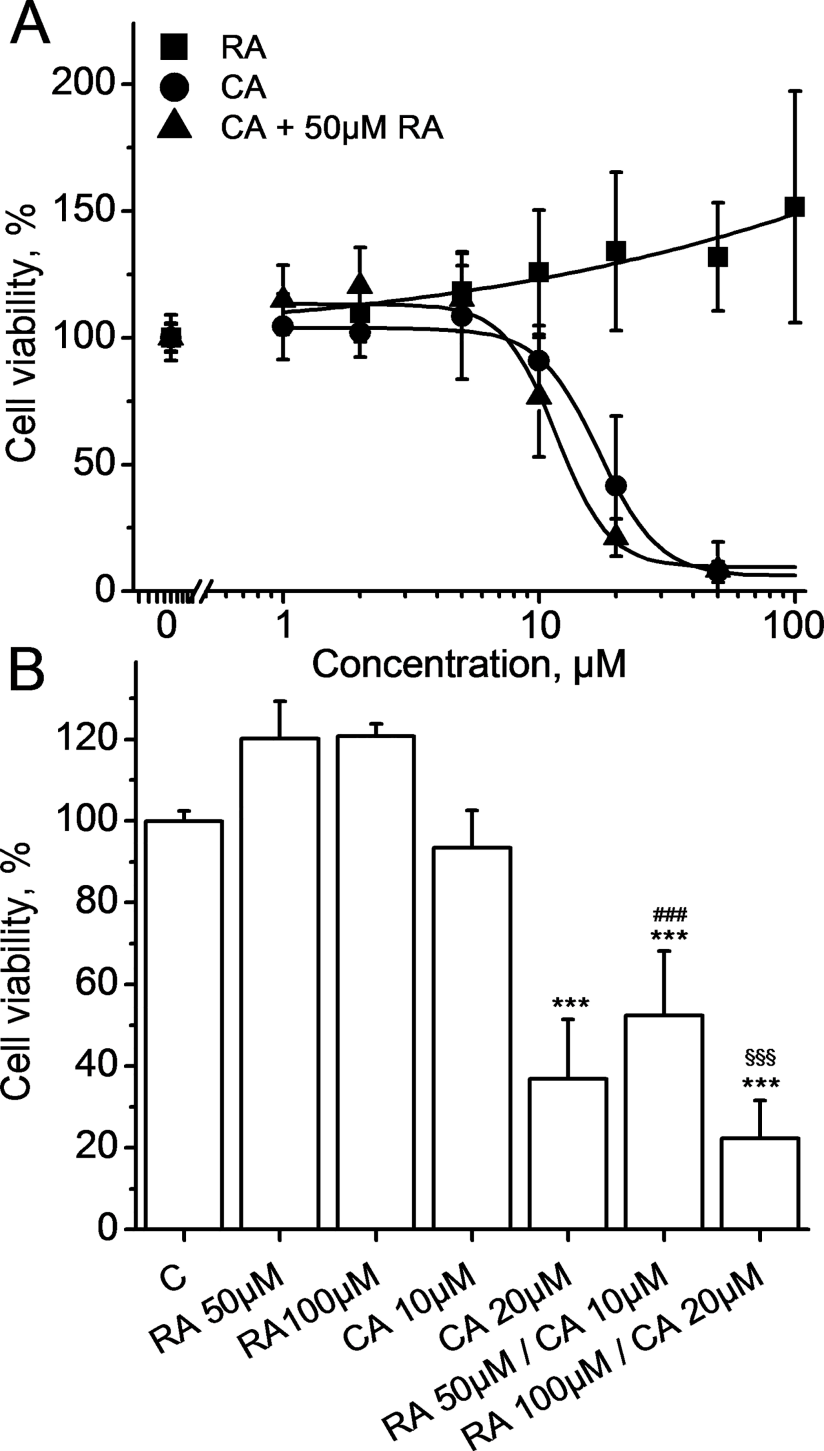
**A. Cell viability dose-response curves for RA alone (squares), CA alone (circles) and CA + 50** μ**M RA (triangles) at 24h.** Data represent Mean±SD of 5 experiments for CA and RA alone and 4 experiments for RA+CA. Lines represent best fit to the logistic equation. **B. Synergy of CA and RA on cellular viability at 24 h.** Data represent Mean±SD of 5 experiments. Cells were treated with RA (at 50 and 100 μM), CA (at 10 and 20 μM) and RA+CA (at 50 and 10μM; 100 and 20μM, respectively). *** p< 0.001 *vs* C, ^###^ p< 0.001 *vs* CA 10 μM, ^$ $ $^ p< 0.001 *vs* CA 20 μM.

As shown in Fig 1A, CA treatment inhibited cell viability in a dose-dependent manner (IC_50_ CA = 17.13±1.06 μM), while RA treatment had no cytotoxic effect on HLF cells. To study the combined effect of these two molecules, we added 50 μM RA to HLF cells treated with different doses of CA. Our results demonstrate that RA enhanced the cytotoxic effect of CA, IC_50_of CA in the presence of 50 μM RA shifted to 11.70±1.46 μM. As shown in Fig 1B, the effect of RA on CA-induced cytotoxicity occurs at different concentrations demonstrating the synergy between these two molecules. Treatment with low cytotoxic concentration of CA (10μM) in the presence of 50 μM RA induced a more important decrease in HLF viability as compared to CA alone (p<0.001). Taken together, these results indicate that CA inhibits viability of HLF cells and this effect can be boosted by the addition of RA.

### Effects of CA, RA, and CA + RA on induction of apoptosis of HLF cells

**Fig 2:**
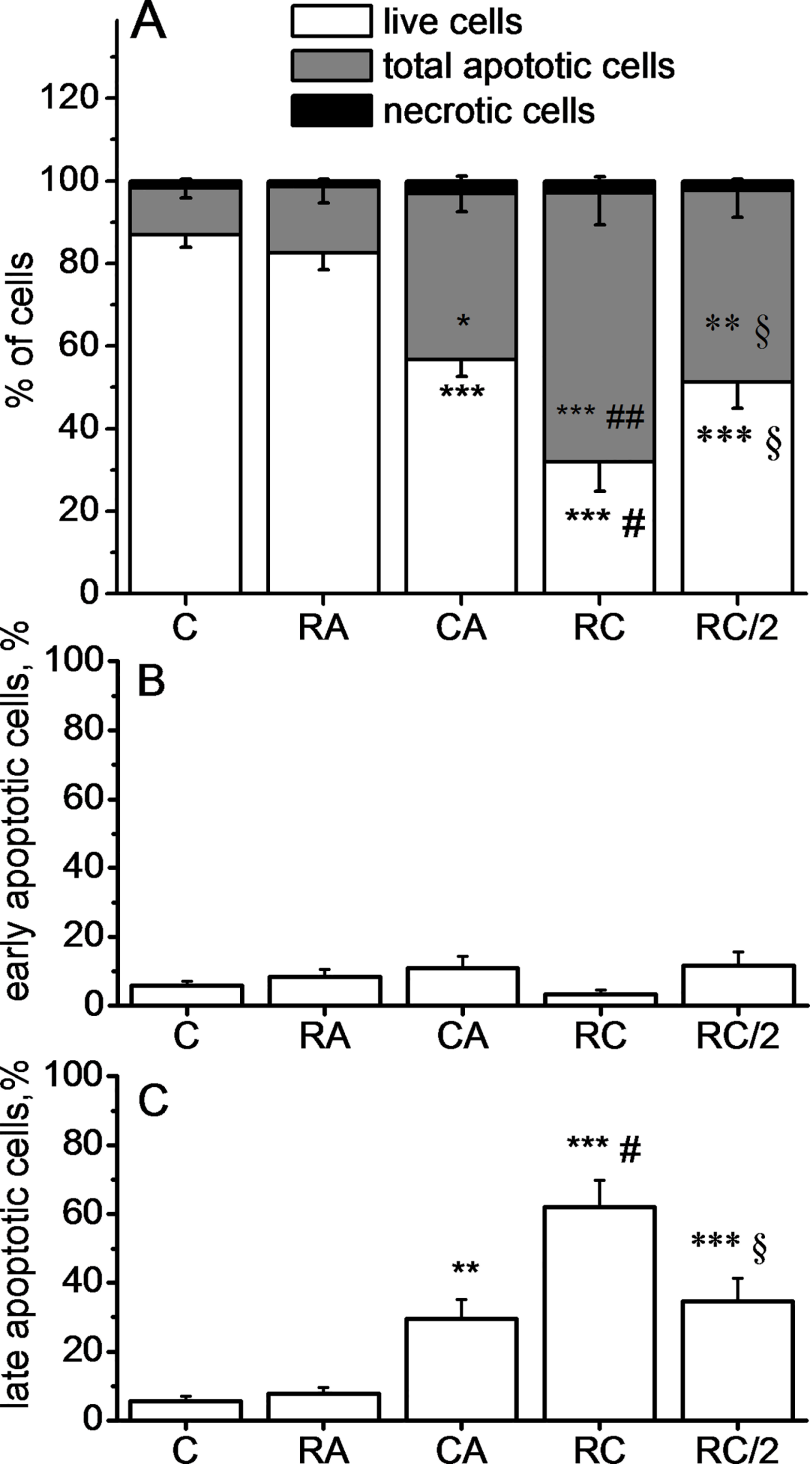
**Quantification of live cells, total apoptotic and necrotic cells (A), early apoptotic cells (B) and late apoptotic cells (C) using annexin V/7AAD staining.** Data represent Mean±SD of 8 experiments. Cells were treated with 100 μM RA, 20 μM CA, 100 μM RA + 20 μM CA (RC) or 50 μM RA + 10 μM CA (RC/2). Data are expressed as the percentage of gated cells. ***p<0.001, **p<0.01 and *p <0.05 *vs*. Control, respectively, ^###^p<0.001 and ^#^p <0.05 *vs* CA alone, ^§^p<0.05 *vs* RC and p>0.05 *vs* CA alone. Significance signs apply to the corresponding graph bar color

Fig 2A shows a significant induction of apoptosis following the treatment by 20 μM CA, whereas treatment with 100μM RA did not induce any change when compared to the control. The combined treatment with 100μM RA and 20μM CA induced a more important decrease of HLF viability as compared with 20 μM CA alone. Treatment with both molecules at a half dose (50μM RA + 10μM CA), induced a decrease in the living cell rate comparable to that recorded for 20 μM CA alone. The percentage of necrotic cells was very low and negligible confirming that the cell death process involved was apoptosis (Fig 2B). Most of the cells were in late apoptosis stage (Fig 2C, 2D and 2E). Finally, in accordance with the cytotoxicity results, the rate of total apoptotic cells treated with 50 μM RA +10 μM CA was almost identical to that of cells treated with 20 μM CA alone.

### Effects of CA, RA, and CA + RA on myofibroblasts cells viability

**Fig 3:**
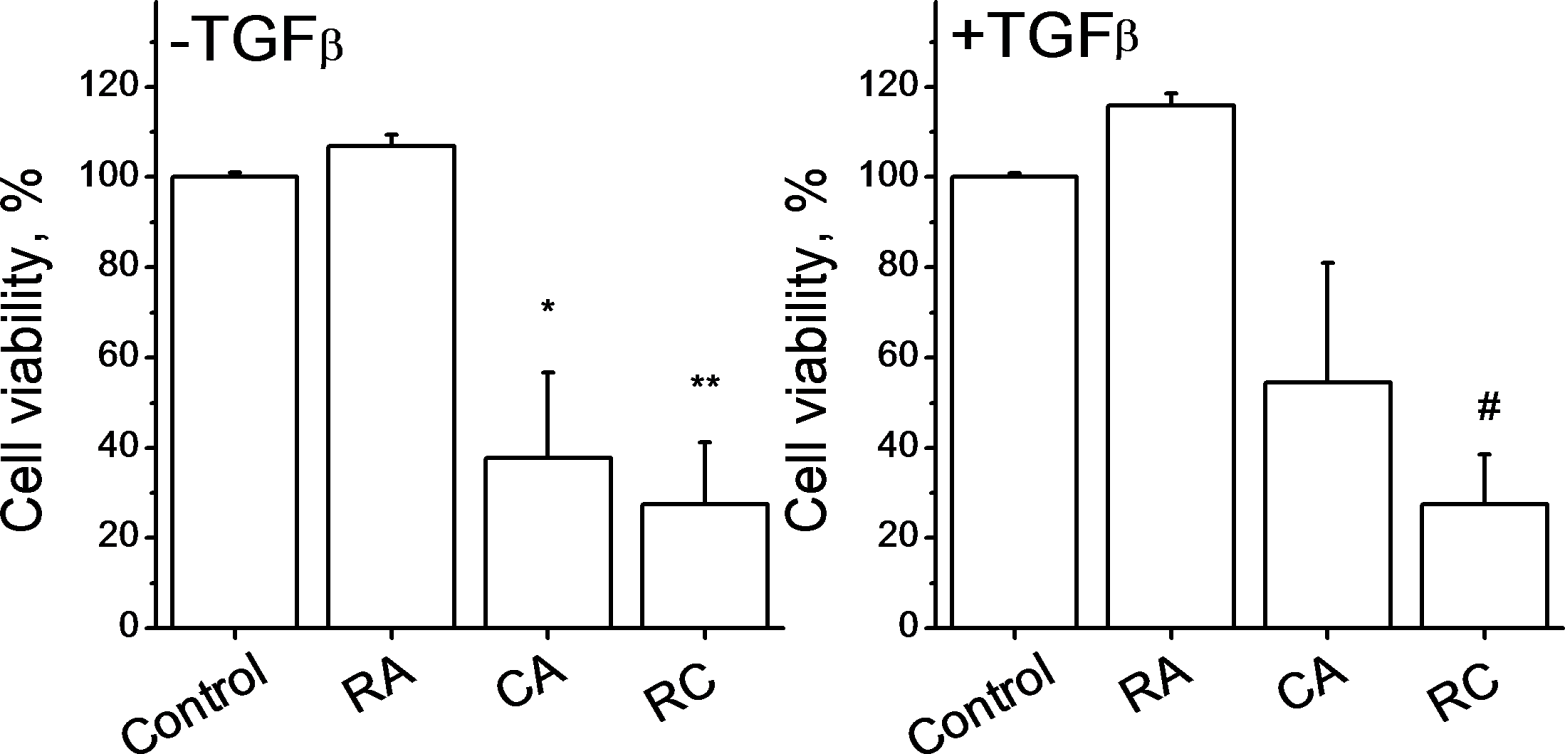
Effects of CA, RA, and CA + RA on viability of myofibroblast cells. Data represent Mean±SD of 3 experiments. HLFcells were incubated with or without TGFβ (2 ng/ml) for 60 hours to induce their differentiation to myofibroblasts. Then cells were treated with 100 μM RA, 20 μM CA and with a combination of 100 μM RA+20 μM CA (RC) for 24 hours. *p<0.05 vs control −TGFβ, **p<0.01 vs control–TGFβ, #p<0.05 vs control +TGFβ.

TGFβ is involved in the induction of fibroblasts differentiation into myofibroblasts and these cells are the most abundant cells in advanced stages of fibrosis. We cultured HLF cells for 60 hours in the absence or in the presence of TGFβ (2ng/ml) in order to induce this trans-differentiation. TGFβ-induced cell transformation has been confirmed by the appearance of stellate cells and cells with bigger number of focal adhesions, as observed under microscope. Our results show that the combination of CA and RA induced a strong decrease in cell viability compared to the control demonstrating that these molecules act in the same manner in both fibroblasts and myofibroblasts (Fig 3).

### Effects of CA, RA, and CA + RA on RLF, alveolar type II cells, L929 cells and A549 cells

**Fig 4:**
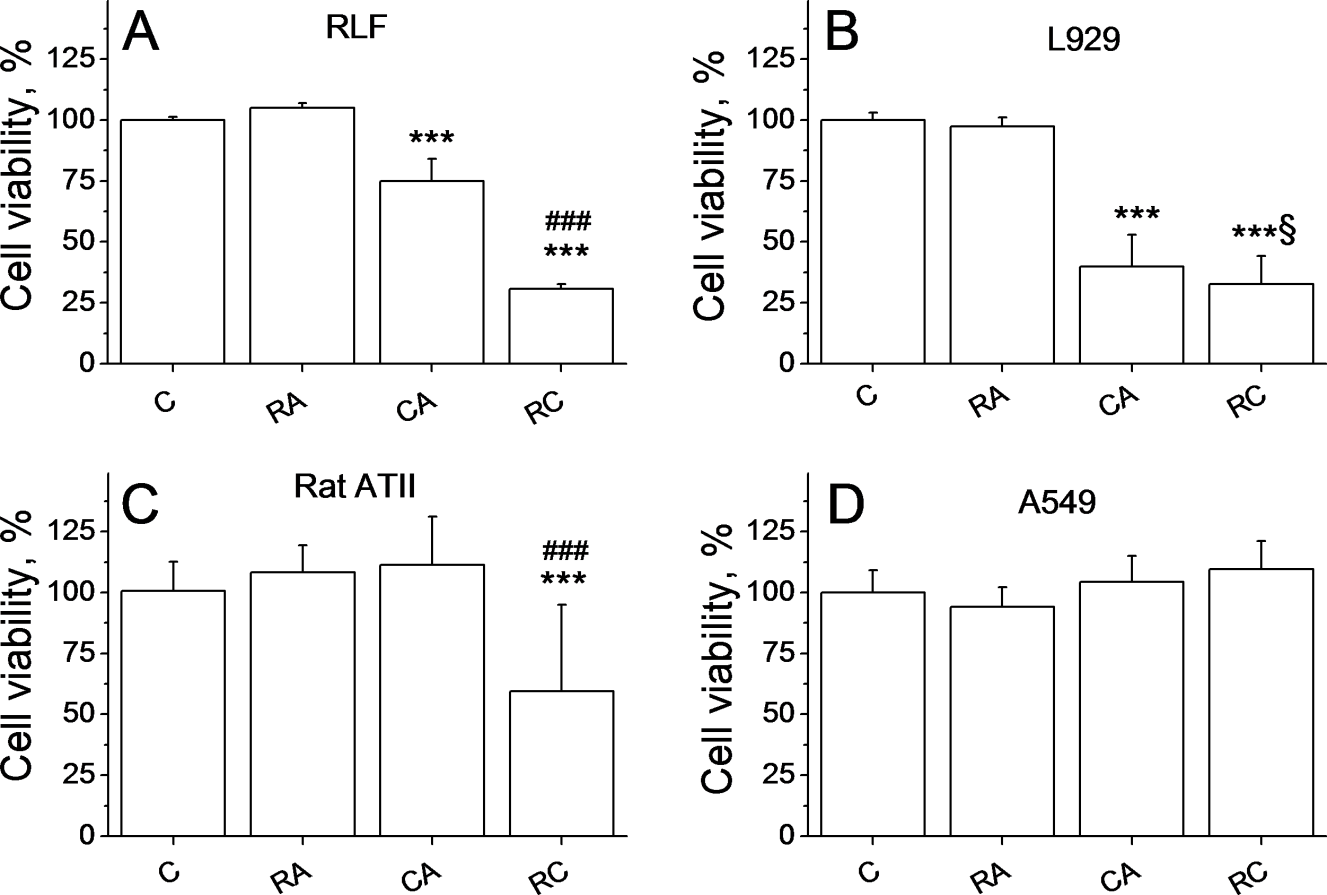
**Effects of CA, RA, and CA + RA on viability of rat lung fibroblasts cells (A), L929 cells (B), rat alveolar Type II cells (C) and A549 cells (D).** Data represent Mean±SD of 5 experiments for each cell type. Cells were treated with 100 μM RA, 20μM CA or with combination of 100 μM RA +20 μM CA (RC) for 24 hours. ***p<0.001 *vs* C, ^###^p <0.001 *vs* CA, ^§^p<0.05 *vs* CA.

As shown in Fig 4A, treatment with 20 μM CA induced a 20% reduction of RLF viability compared to the control (p<0.001), while the combination RA/CA induced a 70% decrease of viability. Fig 4B shows that treatment of L929 fibroblasts (originated from mouse areolar tissue) with 20 μM CA or with 100 μM RA/20 μM CA combination induced a significant reduction of cell viability compared to control (p<0.001 vs control). The combination treatment has, therefore, similar effect in all types of fibroblasts tested: HLF cells, rat lung fibroblasts and L929 cells.

To see whether CA and RA were cytotoxic to other cell types existing on the same territory as fibroblasts, we tested the effect of CA and RA on rat alveolar type II cells and A549 cells. Our results showed that treatment with RA, CA or CA/RA does not affect A549 cells viability (Fig 4D).Treatment with 100 μM RA or 20 μM CA alone had no effect on rat epithelial type II cells, while the combination of two molecules decreased cell viability by 40% (p<0.001 vs control, Fig 4C), but this decrease was significantly lower compared to nearly 80% observed for HLF and RLF.

### Effects of CA, RA, and CA + RA on HLF cell growth and migration at non-cytotoxic concentration

**Fig 5:**
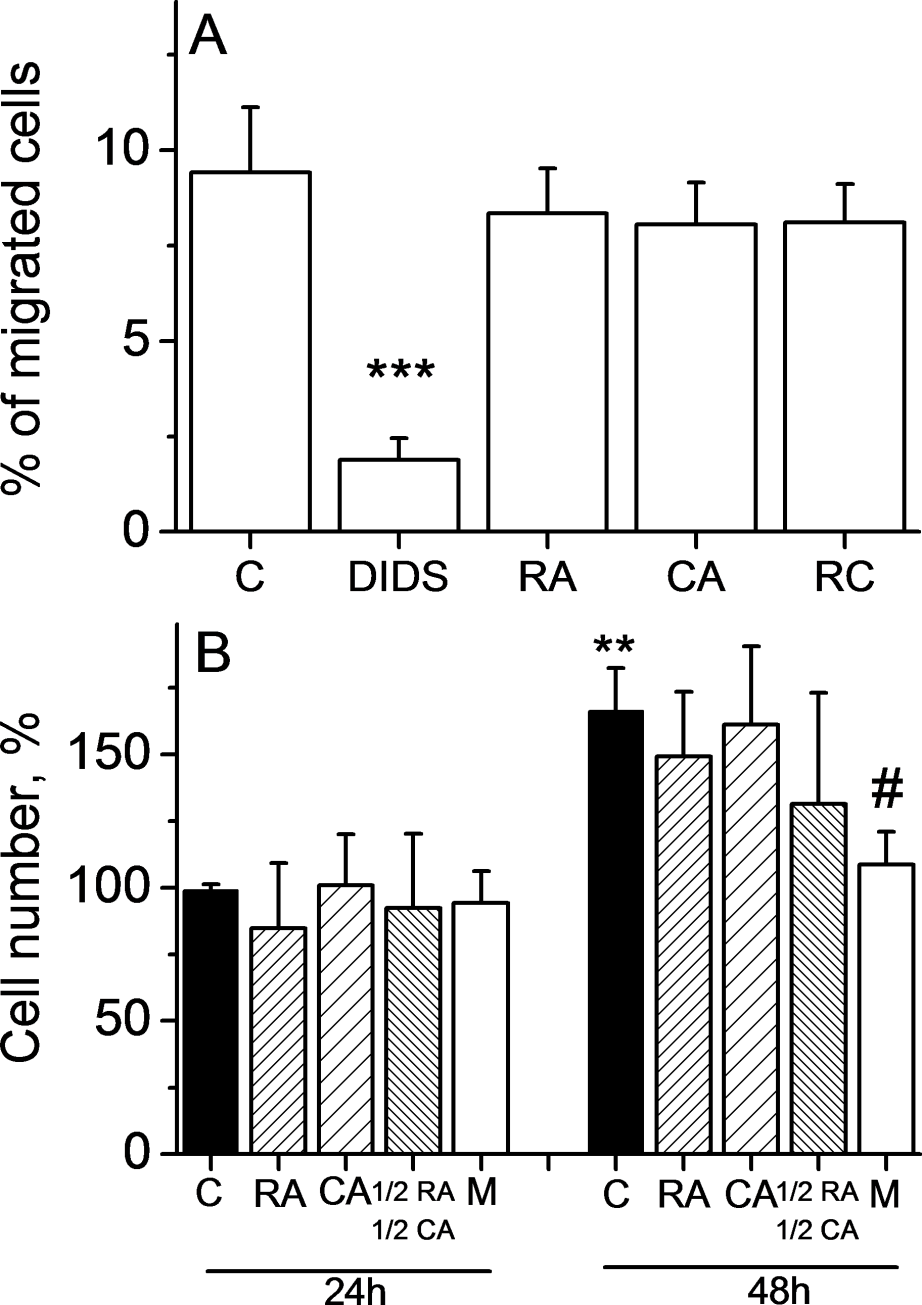
CA, RA, and CA + RA do not affect HLF migration and cell growth at non-cytotoxic concentrations. **A. Cell migration.** Data represent Mean±SD of 3 experiments. Cells were treated with 5 μM RA, 2 μM CA, with combination 2.5 μM RA + 1 μM CA (RC) or 100 μM DIDS. Data represent the percentage of seeded cells after 6 hours of cell migration. ***p<0.001 vs C. **B. Cell growth.** Data represent Mean±SD of 5 experiments. Cells were treated with 5 μM RA, 2 μM CA, with combination 2.5 μM RA + 1 μM CA or with 1.5 μM mitomycin (M) for 24 and 48 hours. Data represent the percentage of viable cells normalized to the control at 24 h. **p = 0.01 *vs* C 24h, ^#^p<0.05 vs C 48h.

In order to see the effect of CA, RA and CA+RA on cell growth and cell migration that is not related to decreased cell viability, we have chosen to study these phenomena at non-cytotoxic concentrations of these substances (5 μM RA and 2 μM CA). Fig 5A displays the results on cell migration and suggests that neither CA, nor RA, nor their combination affected cell motility, while 100 μM DIDS (used as positive control, [28]) significantly inhibited cell migration (p<0.001 vs control). Same non-cytotoxic concentrations had no significant effect on HLF cell growth (Fig 5B). The anti-proliferative effect of 1.5μM mitomycin used as a positive control was visible after 48 h.

### Effects of CA, RA, and CA + RA on HLF cell cycle

**Fig 6:**
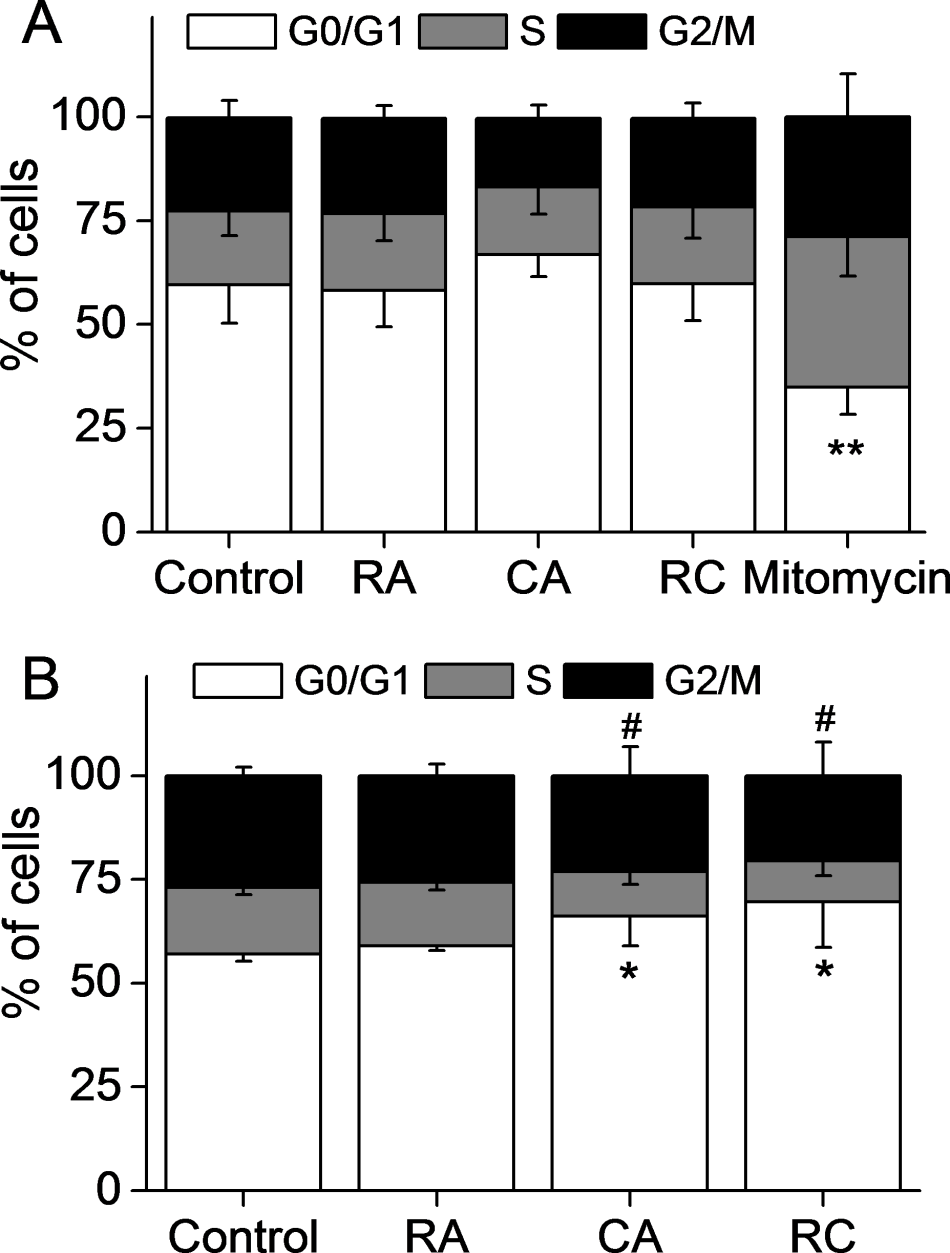
**CA, RA, and CA + RA have no effect on HLF cell cycle at non-cytotoxic concentrations (A) and induce G0/G1 arrest at cytotoxic concentrations(B).** In **A**, cells were treated with 5 μM RA, 2 μM CA, with combination 2.5 μM RA + 1 μM CA (RC) or with 1.5 μM mitomycin (M) for 24 hours. In **B**, cells were treated with 100 μM RA, 20 μM CA or with combination 100 μM RA + 20 μM CA (RC). Data are expressed as the percentage of cells in different cell cycle phases (G0/G1, S and G2/M) and are the mean values μ SD of three (**A**) and four (**B**) different experiments. **p = 0.01 vs C in G0/G1 phase, *p<0.05 vs C in G0/G1 phase, ^#^p<0.05 vs C in S phase.

In accordance with the above results, RA and CA at non-cytotoxic concentrations (5 μM and 2 μM, respectively) did not induce changes in HLF cell cycle (Fig 6A). In positive control, we could observe an accumulation of cells in phase S following treatment with 1.5 μM mitomycin (Fig 6A). CA alone at concentration of 20 μM or in combination with 100μM RA induced accumulation of cells in G0/G1 phases at the expense of cells in other phases, while RA alone had no effect. There was no difference in the effect of CA alone and in combination with RA, which suggests the lack of synergic action of CA and RA at the level of cell cycle arrest in G0/G1 (Fig 6B).

### Western blotting on MAPK-p38, Akt and p21 in HLF cells

**Fig 7:**
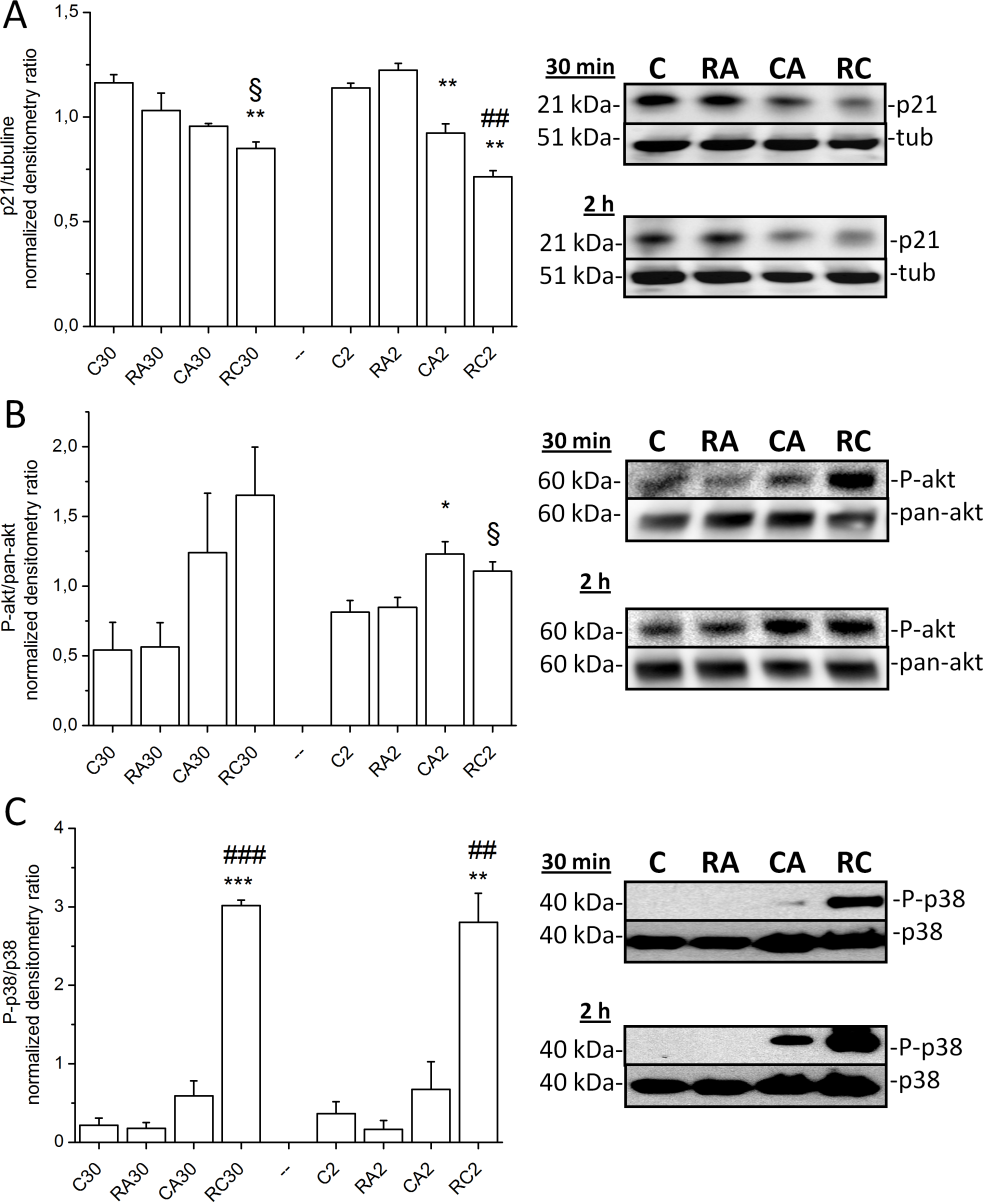
**Effects of CA, RA, and CA + RA on p21 (A), Akt (B) and p-38 (C) activities in HLF cells.** Cells were treated with 100 μM RA, 20 μM CA and with combination 100 μM RA + 20 μM CA (RC) for 30 min and 2 hours. One representative immunoblot image and band densitometry analysis of three experiments for Akt and p21 and four experiments for p38 are shown. Akt and p-38 activities were calculated as a ratio of band optical density between phosphorylated and non phosphorylated protein, while p21 expression was loading normalized using tubulin. Data are expressed as a relative activity normalized to the mean value for each experiment series. ***p<0.001, **p<0.01 and *p<0.05 vs. corresponding Control, respectively, ^###^p<0.001, ^##^p<0.01 vs corresponding CA alone, respectively, ^§^p<0.05 vs CA alone.

MAPK-p38 and Akt were selected for western blotting as the two pathways implicated in cell stress and cell survival, respectively. The p21 (CIP1/WAF1) protein is a potent cyclin-dependent kinase inhibitor and thus functions as a regulator of cell cycle progression at G1 and S phase and it was also chosen for exploration. There was a robust increase in phosphorylated MAPK-p38 levels 30 min and 2 hours after RA+CA exposure (p<0.05 vs control), while CA alone caused significantly smaller increase in phospho-p38 level. Similar results were observed with phospho-Akt after 30 min and 2 hours of RA+CA exposure (Fig 7B and Fig 7C, p<0.05). Our results showed also a progressive decrease in p21 expression after 30 min (p<0.01 vs control) and 2 hours (p<0.001 control) of RA+CA exposure, while CA alone caused smaller p21 attenuation (Fig 7A).

### Effect of pharmacological inhibition of signaling pathways on CA+RA cytotoxicity

**Fig 8:**
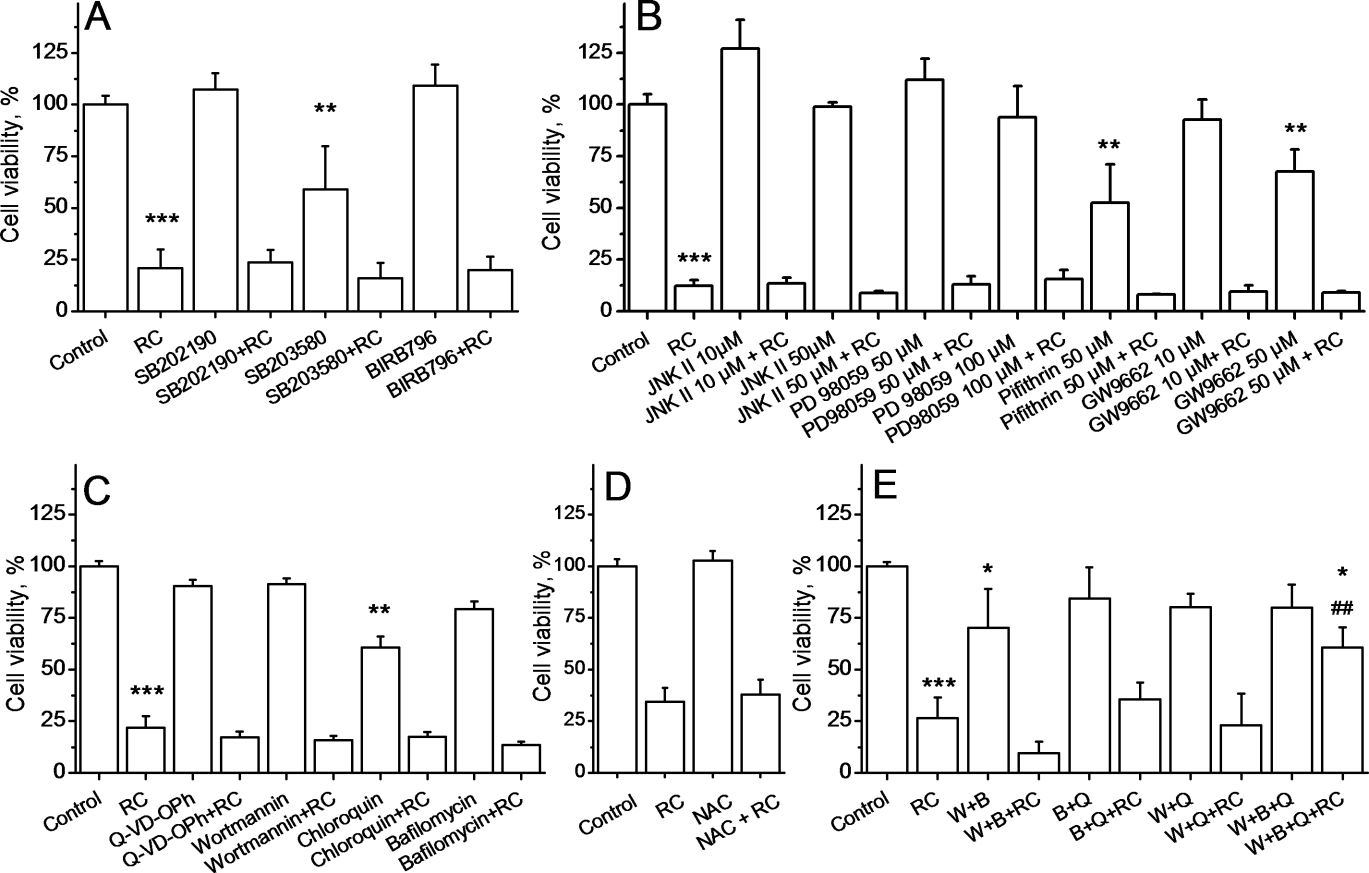
Effect of pharmacological inhibition of signaling pathways on CA+RA cytotoxicity. **A. MAPK inhibitors do not affect CA+RA cytotoxicity in HLF cells. A.** Data represent Mean±SD of 3 experiments. Cells were treated with 10 μM SB202190, 20 μM SB203580 and 10 μM BIRB796 one hour before treatment with 100 μM RA + 20μM CA (RC) for 24h. ***p<0.001 *vs* C, **p<0.05 *vs* C **B. ERK inhibitor PD98059, JNK inhibitor II, p53 inhibitor pifithrin-**μ **and PPARγ inhibitor GW9662 do not affect CA+RA cytotoxicity in HLF cells.** Data represent Mean±SD of 3 experiments. Cells were treated with 10 μM and 50 μM of JNK inhibitor II, 50 μM and 100 μM of PD98059, 50 μM pifithrin-μ, 10 μM and 50 μM GW9662 one hour before treatment with 100 μM RA + 20μM CA (RC) for 24h. **C. Autophagy inhibitors do not affect CA+RA cytotoxicity in HLF cells.** Data represent Mean±SD of 3 experiments. Cells were treated with 1 μM of Q-VD-OPh, 100 nM of wortmannin, 50 μM chloroquin and 100 nM bafilomycin A1 one hour before treatment with 100 μM RA + 20 μM CA (RC) for 24h. ***p< 0.001 *vs* C, **p< 0.05 *vs* C. **D. Antioxidant N-acetyl-cystein does not inhibit CA+RA cytotoxicity in HLF cells.** Data represent Mean±SD of 3 experiments. Cells were treated with 10 mM of N-acetyl-cystein (NAC) one hour before treatment with 100 μM RA + 20 μM CA (RC) for 24h. **E. Combination of p38 inhibitor BIRB796, PI3K/Akt inhibitor wortmannin and pan-caspase inhibitor Q-VD-OPh attenuates cytotoxicity of CA+RA in HLF cells.** Data represent Mean±SD of 3 experiments. Cells were treated with 1 μM of Q-VD-OPh (Q), 100 nM of wortmannin (W) and 10 μM BIRB796 (B) in different combinations one hour before treatment with 100 μM RA + 20 μM CA (RC) for 24h. ***p< 0.001 *vs* C, *p< 0.05 *vs* C, ^##^p<0.01 vs RC.

Our results demonstrate that none of the MAPK-p38 inhibitors used alone (SB202190 at 10 μM, SB203580 at 20 μM and BIRB796 at 10 μM) could prevent cytotoxic effect of CA+RA treatment in HLF (Fig 8A). In addition, SB203580 was slightly cytotoxic and induced a significant decrease in cell viability compared to control. Further, since cell cycle arrest in G0/G1 could be promoted by activation of ERK, we used ERK inhibitor PD98059 at two concentrations (50 and 100 μM). However, it also failed to attenuate cell mortality induced by CA+RA (Fig 8B). Implication of another signaling protein, JNK that promotes apoptosis when activated, was also ruled out using JNK inhibitor II used at two concentrations (10 and 50 μM, Fig 8B). It has been shown that carnosic acid may promote autophagy [29]. Accordingly, we used autophagy inhibitors (100 nM wortmannin, 50 μM chloroquin and 100 nM bafilomycin A1) to see whether they could prevent CA+RA cytotoxicity. As shown in Fig 8C, all of them failed to affect CA+RA action. Chloroquin at the concentration used was found to be slightly cytotoxic. In addition, Fig 8C shows that pan-caspase inhibitor Q-VD-OPH used at 1 μM was ineffective against CA+RA cytotoxicity. Other inhibitors were further tested: 50 μM pifithrin-μ (inhibitor of p53), 10 μM and 50 μM GW9662 (PPARγ inhibitor) and 10 mM N-acetyl-L-cystein (antioxidant). None of these inhibitors could attenuate the effect of CA+RA that rules out the implication of p53, PPARγ and reactive oxygen species generation (Fig 8B and Fig 8D).

Given the lack of the effect of pharmacological inhibition of the signaling pathways on the CA+RA cytotoxicity, we hypothesized that cellular death is induced by several parallel cascades. We have found that only triple combination of pan-caspase inhibitor Q-VD-OPH, BIRB796 and wortmannin was able to attenuate the effect of RC by 53% (p<0.01, Fig 8E). Hence, the protective effect of this triple inhibitor combination was incomplete and suggests that there are other signaling pathways implicated in cytotoxicity induced by carnosic and rosmarinic acids besides p38/PI3K-Akt-autophagy/caspase pathways. It should be also noted that combination of pan-caspase inhibitor separately with BIRB796 or wortmannin did not affect the action of RC on fibroblasts (Fig 8E). These results strongly suggest that carnosic and rosmarinic acid combination induces cell mortality by multiple parallel signaling pathways.

### In vivo antifibrotic effect of CA, RA, CA + RA compared to vitamin E

We used well-established rodent model of lung fibrosis induced by bleomycin to test the antifibrotic properties of CA and RA in comparison with the classical antifibrotic control used in this model–vitamin E [30–33]. Death or clinical signs of RA, CA or Vit E poisoning were not detected in any of the experimental groups during this experiment. Body weight variations of rats are given in Table 1. As expected, we notice a significant body weight loss in BLM group compared to the positive gain in control group, RA, CA, RA+CA and vitamin E groups in the same period. BLM-induced body weight loss was attenuated in BLM+AR group (p<0.05) and reversed in BLM+CA group (p<0.001), BLM+CA+RA group (p<0.001) and in BLM+vitamin E group (p<0.001).

**Table 1:** Rat body weight variations, fibrosis score and lung hydroxyproline content.

Groups	Initial/Final weight (g)	Weight gain (%)	Fibrosis score	Hydroxyproline, mg/g of lung tissue
Control	170.0±1.7/193.6±8.0	+13.9±5.7	0.00±0.00	0.156±0.057
BLM	181.6±11.2/158.5±9.7	-12.7±2.8^###^	3.50±0.30	0.558±0.126^###^
RA	177.5±7.4/212.6±10.5	+19.9±6.8	1.16±0.16	0.309±0.109
BLM/RA	180.6±9.3/169±9.2	-6.2±6.4*	1.83±0.40**	0.289±0.029
CA	175.6±6.3/209.8±6.8	+19.5±5.0	0.33±0.21	0.179±0.051
BLM/CA	181.6±11.5/193.6±7.5	+6.7±4.0***	2.16±0.30*	0.204± 0.024**
RA/CA	176.3±6.5/193.8±7.4	+11.8±6.5	1.33±0.21	0.224±0.042
BLM/RA/CA	187.8±5.4/199.3±8.5	+6.1±4.5***	1.50±0.34***	0.163±0.035**
Vit E	166.1±10.8/190.6±6.5	+14.9±5.3	0.83±0.16	0.155±0.034
BLM/Vit E	161.6±8.8/170.1±9.8	+5.3±2.3***	2.00±0.25*	0.181±0.017*

Data are expressed as Means±SD (n = 8)

^*###*^*p<0*.*001 vs* C

***p<0.*001 vs* BLM

**p <0.*01 vs* BLM

*p < 0.*05 vs* BLM.

**Fig 9:**
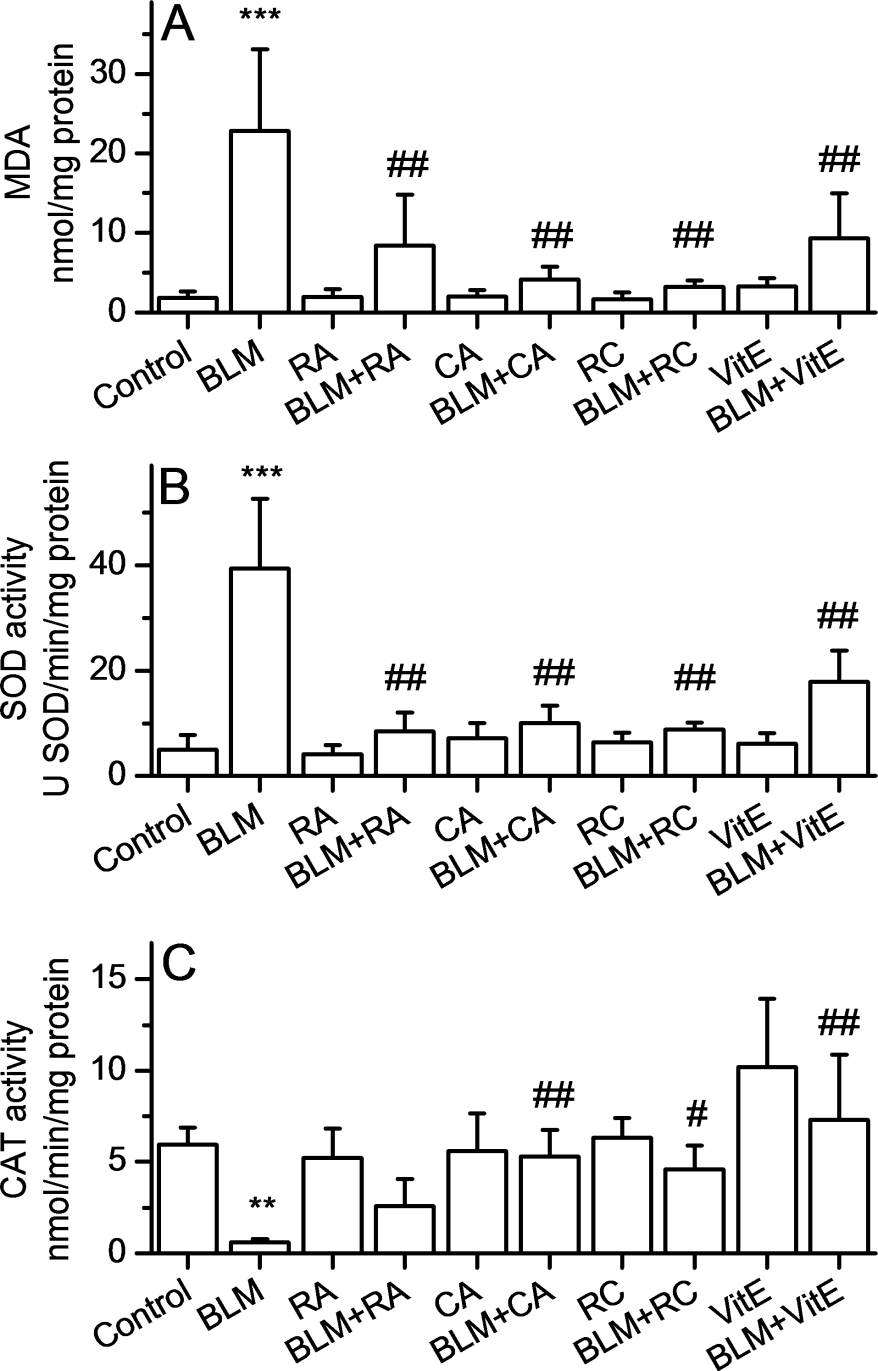
RA, CA, RA+CA and Vitamin E alleviate bleomycin-induced oxidative stress in lung tissue. **A. Effect of RA, CA, RA+CA and Vit E on bleomycin-induced lipid peroxidation.** Malondialdehyde levels represent Mean±SD (n = 8) ***p<0.*001 vs* C, ^*##*^*p<0*.*01 vs* BLM.**B. Effect of RA, CA, RA+CA and Vit E on bleomycin-induced changes in SOD activity.** Results are expressed as Mean±SD (n = 8) ***p<0.*001 vs* C, ^*##*^*p<0*.*01 vs* BLM.**C. Effect of RA, CA, RA+CA and Vit E on bleomycin-induced changes in catalase activity.** Results are expressed as Mean±SD (n = 8) **p<0.*01 vs* C, ^*#*^*p<0*.*05 vs* BLM, ^*##*^*p<0*.*01 vs* BLM.

Lung lipid peroxidation was assessed by malondialdehyde (MDA) determination and the results are shown in Fig 9A. MDA lung level is significantly higher in the BLM group compared to the control group (p<0.001). All types of curative treatments induced significant decreases in MDA level compared to BLM group (p<0.001). Moreover, the combined treatment with BLM+CA+RA showed cumulative antioxidant effect compared to BLM+RA and BLM+CA treatment. Interestingly, the combined curative treatment with RA+CA or with CA alone appear to be more potent in reducing MDA level compared to curative treatment with vitamin E.

The activity of superoxide dismutase (SOD) in the lung tissue, as shown in Fig 9B, was significantly increased by BLM treatment when compared with control (p<0.001). We observed a significant reduction in SOD activity following treatment with BLM+RA, BLM+CA, BLM+CA+RA and BLM+VitE compared to BLM group. Again, the effect of vitamin E on normalization of SOD activity appears to be less potent than that of RA, CA or RA+CA.

The activity of catalase in the lung tissue, as shown in Fig 9C, was significantly reduced by BLM treatment when compared with control (p = 0.0015). In BLM+RA group, we did not notice significant restoration of catalase activity compared to BLM group (p>0.05), while near full restoration of this level was observed in BLM+CA and BLM+CA+RA groups. Finally, curative treatment with vitamin E induced an increase in catalase level compared to BLM group (p<0.001) but always to a lesser degree compared with the curative treatments with CA or RA+CA.

**Fig 10:**
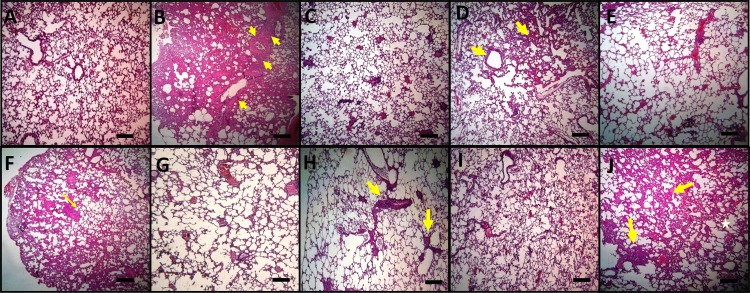
Histopathological findings (H&E, ×100) in lung tissue. **(A)** Control **(B)** BLM, arrows indicate a thickening of interalveolar septum and formation of fibrous mass, **(C)** RA **(D)** BLM+RA and (**F)** BLM+CA, arrows indicate interstitial thickening and of alveolar walls, **(E)** CA, **(G)** CA+RA, **(H)** BLM+RA+CA arrows indicate some fibrousmass without an apparent impairment to lung architecture, **(I)** Vit E **(J)** Vit E+BLM arrows indicate a thickening of interalveolar septum and formation of fibrous mass, asterisk indicate severe inflammation with presence of inflammatory cells. One representative example is shown for each group. Scale bar = 150 μm.

**Fig 11:**
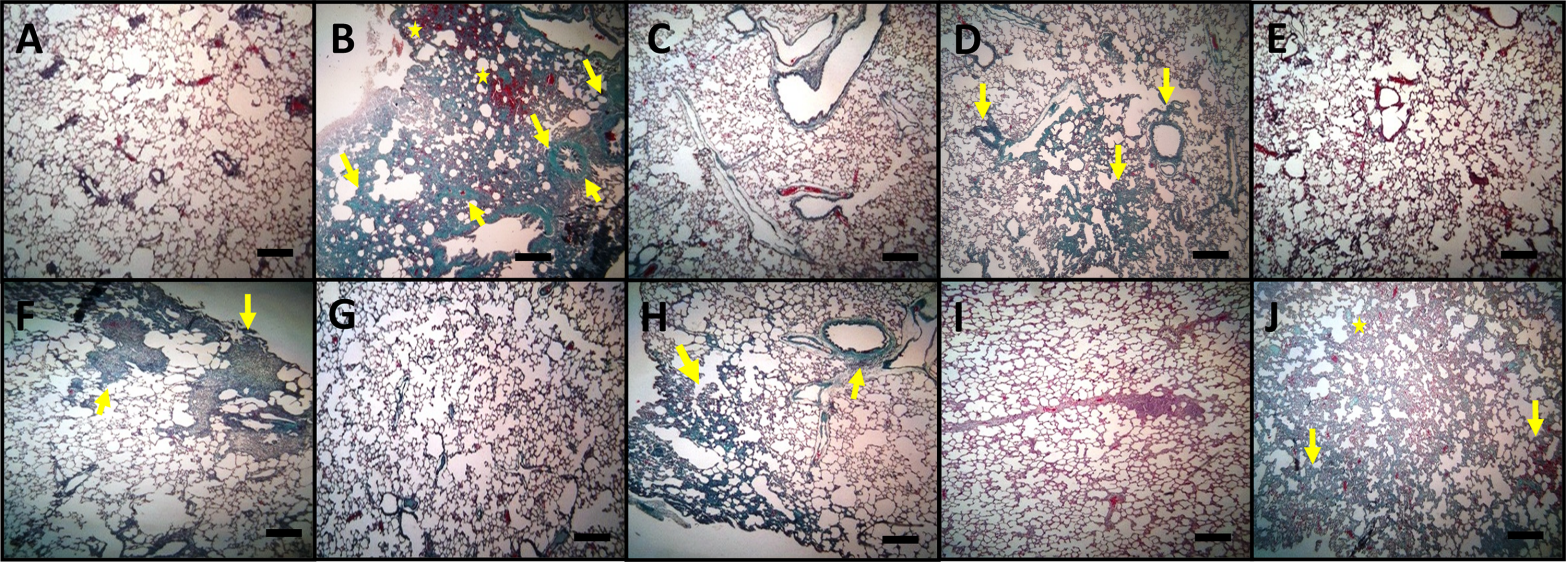
Histopathological findings (Masson’s trichrome, ×100) in lung tissue. **(A)** Control **(B)** BLM, **(C)** RA **(D)** BLM+RA **(E)** CA,(**F)** BLM+CA, **(G)** CA+RA, **(H)** BLM+RA+CA **(I)** Vit E **(J)** Vit E+BLM. Arrows indicate condensed bundles of collagen recognized in green color. Asterisks indicate severe inflammation with presence of inflammatory cells. One representative example is shown for each group. Scale bar = 150 μm.

Fig 10 displays histopathological findings in lung tissue stained with hematoxylin-eosin. Lungs from rats treated with BLM showed an architectural damage with a restricted alveolar space, a thickening of interalveolar septum and formation of fibrous mass (arrow) accompanied by severe inflammation (Fig 10B). Lungs from rats treated with BLM+RA (Fig 10D) showed a moderate thickening of alveolar walls (arrow) and a decrease in fibrotic bands without an apparent damage of lung architecture, while lungs of rats treated with BLM+CA (Fig 10F) showed a decrease in the interstitial thickening and in fibrotic bands (arrow). Moreover, treatment with BLM+CA+RA (Fig 10H) showed a significant decrease in fibrotic bands when compared to BLM+RA and BLM+CA groups. Finally lungs from rats treated with BLM+VitE (Fig 10J) showed an architectural damage with a thickening of interalveolar septum and the formation of fibrotic bands (arrows), accompanied by severe inflammation (asterisk). Fig 11 displays histopathological findings in lung tissue stained with Masson’s trichrome. This staining showed condensed bundles of collagen in BLM group (Fig 11B) recognized in green (arrows) and a severe inflammation recognized by the infiltration of red inflammatory cells colored in red (stars). Treatment with BLM+RA (Fig 11D), BLM+CA (Fig 11F) and BLM+CA+RA (Fig 11H) resulted in less abundant bands of collagen (arrow) compared to BLM group. Treatment with BLM+VitE (Fig 11J) showed no improvement in lung histology compared to BLM group—we still observed dense bundles of collagen (arrow).

The gravity of fibrosis was estimated through the use the semi quantitative grading system and results are presented in Table 1. Fibrosis score in BLM+RA, BLM+CA, BLM+CA+RA and vitamin E+BLM groups were significantly decreased compared to BLM group. Antifibrotic effect was further corroborated using lung hydroxyproline quantification that followed fibrosis score (Table 1).

## Discussion

In our previous work, we have demonstrated that rosemary extract, which is rich in rosmarinic and carnosic acids, was able not only to attenuate bleomycin-induced pulmonary fibrosis but also to cure it, when they were given after fibrotic remodeling took place [17]. We suggested that rosmarinic and carnosic acids modulate cellular dynamics in lungs during fibrosis progression, which reinforces the anti-fibrotic property of this plant extract [17]. Our present results clearly indicate that the combined application of carnosic and rosmarinic acids has synergic pro-apoptotic effect in fibroblasts (rat and human lung fibroblasts and L929 cells) and in TGFβ-transformed fibroblasts only. The lung epithelial cells are either insensitive to this treatment (A549 cells) or subject to death at significantly lower rate (AT II cells). The primary cause of this cellular death is apoptosis. There was no synergic effect of these two molecules on the induction of cell cycle arrest, which suggests that cell cycle modulation is owed to CA only and is not related to the synergic pro-apoptotic effect. At the *in vivo* level, carnosic and rosmarinic acids were both able to reverse lung architecture disorganization and to decrease overall oxidative stress induced by BLM. It should be noted that while they were both equally effective on the oxidative stress normalization, CA and CA+RA were more efficient in decreasing fibrosis score and collagen deposition.

BLM is known inductor of experimental pulmonary fibrosis causing oxidative injury in lungs [34]. The cytotoxicity of BLM is determined by the induction of free radicals, which in turn cause DNA strand breakage [35], protein and lipid decomposition and the disruption of enzyme activity [36]. The intratracheal instillation of BLM causes damage to alveolar epithelial cells, followed by the development of neutrophilic and lymphocytic pan-alveolitis within the first week [20]. Consequent activation of profibrotic tissue repair results in lung architecture damage due to collagen overproduction. To counteract BLM-induced pulmonary fibrosis, we have employed as a positive control known antioxidant–vitamin E [30–33]. Despite the normalization of the oxidative stress, vitamin E was less efficient in the reversion of changes in lung structure in contrast to CA and RA. Unfortunately, the dose CA that we have chosen for the in vivo study was high enough to exert strong anti-fibrotic effect alone, so that the observation of the *in vivo* synergy between RA and CA requires further study using much lower doses. Our results, nevertheless, strongly suggest that CA and RA are the major actors of the *in vivo* antioxidant and anti-fibrotic effects of rosemary extract [17].

The interactions between alveolar epithelial cells and fibroblasts contribute to the development of pulmonary fibrosis [37,38]. In our study, the estimated half-cytotoxicity concentration value of CA+RA for rat alveolar epithelial cells was at least twice bigger than that for fibroblasts. Accordingly, careful choice of the dose may ensure that no epithelial cells are affected, while fibroblasts and myofibroblasts continue to disappear. Myofibroblasts are originated from resident fibroblasts [2] and their abundance correlates with the pulmonary fibrosis progression [39]. Moreover, at the stage of recovery from profibrotic insult, it might even constitute an advantage to eliminate injured epithelial cells as well. This is because these cells secrete pro-fibrotic factors under these conditions, including TGF-β that secreted by alveolar epithelial cells. Its activity is characterized by the promotion of the production of the extracellular matrix [38], the differentiation of fibroblasts into myofibroblasts [40,41], and the inhibition of autophagy in fibroblasts [42]. Increased viability of myofibroblasts could explain the transition of a normal lung repair process into a fibrogenic one [43]. However, more experiments are necessary to see whether these molecules induce stronger apoptosis in injured epithelial cells that are implicated in fibrotic process, or this weak effect is restricted only to healthy epithelial cells.

A cyclin-dependent kinase inhibitor p21 regulates cell cycle progression by causing an arrest in G1 to S phase transition [44]. Our results demonstrated G0/G1 phase arrest in cells treated with CA and CA+RA compared to control cells and cells treated with RA alone. The examination of p21 expression showed an inhibition of p21 level after 30 min and 2h of CA and CA+RA treatment. Clearly, the observation of G0/G1 cell cycle arrest conflicts with the disappearance of p21 expression in RA+CA treated cells. However, it has been demonstrated that p21 plays a crucial anti-apoptotic role [45–48]. In our case, HLF cells underwent apoptosis in response to CA+RA treatment and these cells expressed low level of p21 and this loss of p21 could eventually potentiate the apoptotic response. In any case, modulation of cell cycle progression appears to be the effect of CA-treatment only and it is not related to the synergy in the apoptosis induction.

Apoptosis is a natural physiological process of death eliminating unwanted cells during development and maintaining tissue homeostasis. It can also occur in pathological conditions and under intense stress. Mitogen activated protein kinase (MAPK) family members are among many signaling pathways that respond to stress and control other complex programs, such as embryogenesis, differentiation, proliferation and programmed cell death [49–51]. On the other hand, the serine/threonine kinase Akt signaling pathway is also implicated in cell survival, proliferation and apoptosis [52]. Akt pathway activation provides cells the capacity to resist the apoptotic stimuli [53], however it may also witness initialization of autophagy [29]. Our results indicate early activation of MAPK p38 and AKT after 30 min and 2 hours of exposure with CA alone or combined with RA.Moreover, pharmacological inhibitors of a number of signaling pathways (including MAPK p38 and AKT) were ineffective in preventing CA+RA induced cytotoxicity when tested alone. We hypothesized that cellular death is induced by several parallel cascades, which was confirmed by the observationthat triple combination of inhibitors (pan-caspase inhibitor, p38 inhibitor and PI3K/Akt/autophagy inhibitor) could partially attenuate the effect of RA+CA on HLF. The above results considered together indicate that rapid activation of p38 and Akt after 30 minutes and 2h of RA+CA exposure describe simultaneous triggering of MAPK-dependent and autophagy-related cellular death, which occurs via both caspase-dependent and caspase-independent manner [29,45–52]. This also indicates that there are other signaling pathways implicated in cytotoxicity induced by carnosic and rosmarinic acids besides p38/PI3K-Akt-autophagy/caspase pathways. Our observations strongly suggest that carnosic and rosmarinic acid combination induces cell mortality by multiple signaling pathways with none of them being limiting. It means that when one apoptosis-inducing pathway is inhibited, cell death occurs via parallel signaling. Similar pleiotropic cytotoxic action has been reported for other naturally occurring substances, such as curcumin [54] ursolic acid [55], icariside II [56] and diallyl sulfide [57]. Our results are also generally consistent with the published studies using carnosic acid that demonstrated modulation of MAPK p38, PI3K/Akt pathways and activation of both caspase-dependent and caspase-independent signalings [58].

All together, our results demonstrate that strong anti-fibrotic effect of CA and RA is a reflection of the induction of fibroblasts apoptosis in addition to the antioxidant effect of these compounds. Cytotoxicity of carnosic acid in the presence of rosmarinic acid seems to be a complex phenomenon affecting several parallel signaling pathways. Clearly, further studies with the use of inhibitor combinations are needed to identify all the pathways leading to apoptosis. With regard to the mechanism of synergy between carnosic and rosmarinic acid, several scenarios are plausible. First is that these molecules activate distinct pathways that converge at some point of apoptosis induction, however the fact that rosmarinic acid has no cytotoxicity *per se* roules out this scenario. Second, one could expect a chemical recombination between these molecules so that the generated adduct has higher stability in solution and may act continuously over 24 hours, while it is known that carnosic acids is very labile. Finally, this adduct may have distinct cytotoxicity profile and the mechanism of apoptosis induction changes over 24 hours of treatment. Our observation that epithelial cells are less sensitive to carnosic acid and to the combination with rosmarinic acid favors the latter scenario. Moreover, our preliminary experiments using Nuclear Magnetic Resonance spectroscopy indicate a formation of two recombinant products of these molecules. First product appears rapidly upon the contact of rosmarinic and carnosic acid, while the other is generated progressively over 24h period. These two recombinant products are not yet present in chemical databases and their isolation, eventual synthesis and characterization forms basis for our further study.

## Conclusion

Overall, rosmarinic acid plays a potent role as an adjuvant enhancing carnosic acid—induced apoptosis of lung fibroblasts and myofibroblasts and thus this might form a synergic concept for a novel anti-fibrotic therapy.
